# The Institutional Worker

**Published:** 1919-11-29

**Authors:** 


THE
### INSTITUTIONAL WORKER
**Being a Special Supplement to "The Hospital"**

### OUR BUREAU OF INFORMATION.

### Rules for Correspondents.

1. Every letter must be accompanied by the coupon to be cut from the back cover (inside page) of The Hospital, current issue, and must contain the name and address of the correspondent with pseudonym for publication if desired. Replies by post cannot be given save under exceptional circumstances at the Editor's discretion.

2. Letters from Approved Homes in reply to special needs published in the Bureau should state terms and full particulars, and be sent prepaid under cover to the Editor of the Bureau with name written across coupon for identification.

3. Proprietors of Homes which have not yet been entered on the List of Approved Homes, but have spare accommodation likely to suit special needs, are invited to write for an application form for registration. The fee for registration, which includes two announcements of the Home in the Bureau and other privileges, is 10s.

4. All communications to be addressed to the Editor of The Hospital, 28 Southampton Street, Strand, London, W.C. 2, and marked "Bureau of Information."

### INSTITUTION FACTS AND FIGURES.

### The Question Box.

### A CONVALESCENT HOME TIME-TABLE.

The question was :

**Draw up a daily programme with suitable suggestions regarding exercise, light work, amusements, rest hours and diet to be adopted in a Convalescent Home for working girls situated in the country, and occupied mainly by severe cases of anæmia and debility. The ages of the girls vary from 13 to 30, and as the staff consists of matron and cook-general only, each patient, unless confined to bed, is expected to help with light household tasks. The average number of patients is 12. A plentiful supply of milk and eggs is available.**

"Wales" replies :

Meals should be regular, and served at stated times. Patients should rise at 7 a.m., and the following times are suggested for meals :— Breakfast, 7.30 a.m. ; lunch, 10.30 a.m. ; dinner, 1 p.m. ; tea, 4.30 p.m. ; supper, 7.30 p.m.

Diet.

This should be liberal, some of the patients will require more milk and want feeding up; in such cases it is advisable for them to have a glass of hot milk before rising; this could be got ready by the cook as soon as possible in the morning.

Breakfasts.—Should consist of porridge daily, with plenty of new milk. Eggs, bacon, fish alternately; cold boiled ham once a week; devilled kidneys or sweetbreads for a change; coffee made with milk in preference to tea. The patients should not be encouraged to drink much tea.

Lunch.—A glass of milk, hot or cold, biscuits.

Dinner.—Soup, meat, two vegetables, light puddings.

Tea.—A fresh boiled egg, salad in season, jam, or cake.

Supper.—Made-up dishes, such as rissoles, fish cakes, shepherds pie, mince and rice, tripe, cauliflower and cheese, macaroni cheese, boiled onions, etc., cocoa.

The chief aim should be to vary the diet, not to have a repetition often, and not to order the same joint each time. Chops and tomatoes or tomatoe sauce make a nice change for a dinner with chip potatoes.

Light Duty.

From 8 to 10.30 a.m., patients to make their own beds after breakfast, at 8.15 to be ready to help with household work. One or two, according to the number of patients, to wash up, clean the silver, etc. Two to clean the dining-room and sitting-room. Four to do bedrooms, dust staircase, and bathroom. Two to help cook with vegetables, etc. Patients in dining-hall to lay table.

Exercise.

If the weather is fine the patients should go out walking from 10.45 to 1 p.m., if able to do so; otherwise a short walk and rest in garden. After dinner a sleep and rest until 3 p.m., then a quiet game of bowls or croquet, or weeding the garden as a pastime until tea.

After tea another walk, or games, fancywork or sewing out of doors when fine, until supper-time. Bed at 8.30.

Blackberrying when in season is a useful as well as a pleasant pastime. Keeping poultry and rabbits would be another occupation, and a profitable one for the home.

The programme would vary according to the time of the year—in winter more walking would have to be done in the afternoon.

The sewing and mending of the home could be done in the evenings and on wet days. The girls should be encouraged to do sewing for themselves; also fancywork, care being taken that they spend as much time out of doors as possible

Amusements.

Plenty of indoor games should be arranged. A piano and some music are acceptable : some of the patients may be able to play and sing, and thus help the passing of many a pleasant evening. A good library should be got together, for a patient will often read when not feeling inclined for any other pastime.

### REGULATING A PATIENT'S VISITORS.

The question was :

Elaborate a plan whereby the number of patients' visitors may be regulated on visiting days, so that none may be standing about the corridors and all may see the patient in turn. Each patient to be allowed six visitors, but not more than two at a time.

B. R. replies :

An effectual method of allowing six friends to visit a patient—not more than two at a time—is the following :

Assuming the visiting time allowed on any day to be two hours, say from 2 p.m. to 4 p.m., a perforated card, divided into three portions, is issued to the friends of the patient upon admission, the sections being marked A, B, C respectively.

"A" is printed with instructions, stating that the portion is available for two friends to visit the patient from 2 p.m. to 2.40 p.m.

"B" is similarly printed, but with the time stated as 2.40 p.m. to 3.20 p.m.

"C" with the time as 3.20 p.m. to 4 p.m.

The onus of correctly using the various portions rests on the friends—*e.g.*, if there are only two, all three portions of the card would be retained by them; if four, then two of the visitors might hold two portions.

The usual procedure of letting visitors know that time is over would be followed in the hospital, with the exception that the signal would be given at three times—*viz.*, 2.40, 3.10, and 4 p.m. for those holding one section of a visitor's card—those holding a greater number, of course, being permitted to remain longer if they so desired.

### Question for November.

### Reducing Hours without Increasing Staff.

**State how you would deal with the problem of reducing the weekly hours of the Male Manual Staff of a Hospital from say 60 to 48 without increasing the number of such Employees. There are 6 Porters, 2 Boilermen, 1 Laundryman, 3 Gardeners (one of whom assists inside), and a Boy. The Hospital is built upon the pavilion plan, has long corridors (glass roofs and sides) and large grounds. All window-cleaning is done by the Porters. There are 2 steam and 4 hot-water boilers, the Heating System not being central. 187 beds.**

**A minimum payment of 10s. 6d. will be made for each published answer.)**

### RULES.

The following rules must be observed:—

1. Contributions must be written on one side of the paper. Conciseness and terseness are desirable features. The MS. must bear the name and address of the sender and be accompanied by coupon to be cut from the back cover (inside page) of the current issue of THE HOSPITAL. A pseudonym must be chosen if the name is not to be published.
2. Contributions must be addressed to the Editor of THE HOSPITAL Institutional Worker Supplement, 28 & 29 Southampton Street, Strand, London, W.C. 2, should reach him before the end of the current month, and be marked in the left-hand corner "Facts and Figures."

### QUESTIONS INVITED.

IN connection with our Question Box, we offer every month five shillings each for the best questions which are sent in for consideration. The questions may be on any institutional subject and concern any department, from the secretary's to the porter's, or the matron's to the domestic staff; the one essential is that they must be practical and deal with points that have a definite relation to hospital or institutional administration, upkeep, finance, or management. Points relating to artificers' work, housekeeping, the laundry, out-patients, and other practical matters will be welcome. It is hoped that thus, when difficult or doubtful points arise in the course of their work, institutional workers will be encouraged to put them in the form of a question and send them to the Editor, THE HOSPITAL Bureau of Information, 28 & 29 Southampton Street, Strand, London, W.C. 2, marked "Question Box."

The best questions will be published in due course and our readers will be given the opportunity of answering them. Thus all institutional workers may be able to co-operate with the view of helping the work and smoothing the difficulties of each other. In short, we want the Question Box of the *Institutional Worker* to be freely used by every worker habitually.

### ENQUIRIES AND ANSWERS.

### *THE SICK AND IN NEED.*

### Maternity Home—Small Weekly Fee.

WE suggest that you apply to the Matron of the Queen Mary's Maternity Home, Cedar Lawn, North End Road, Hampstead, N.W., for admission to the home. It will be wise to send in your application without delay. The home, which has recently been opened, is for the benefit of the wives and children of men who are, or who have been, serving with His Majesty's Forces; patients who are in a position to do so are required to pay a weekly fee according to their means. — SOLDIER'S WIFE.

### Special Training for Demobilised Nurse.

As you are in receipt of a pension you should apply to the Director of the Training Section, Ministry of Labour, St. Ermin's Hotel, London, S.W. 1, or the King's Fund for the Disabled, Millbank House, Westminster, S.W., for assistance in obtaining the special training you require.—K. E. A.

### *EMPLOYMENT AND TRAINING.*

### Nursing Institutions in London

WE presume that you refer to private nursing institutions. The following is a short list of such institutions in London: Fitzroy House Nurses' Co-operation and Home, Fitzroy Square, W.; Nurses' Co-operation, 22 Langham Street, W.; Mildmay Nursing House, 9 and 10 Newington Green, N.; Auxiliary Nurses' Society, 10 Orchard Street, W. Write to the Lady Superintendent in each case and ask if there is a vacancy on their staff. We regret we cannot give postal replies; a coupon should be enclosed with each inquiry.—E. F. G.

### Hospital for Men.

YOU would no doubt be accepted for training at the Seamen's Hospital, Greenwich, S.E. The training extends over a period of four years, which includes one year in a London hospital for women and children, or you might become a paying probationer at the same institution for twelve months; the premium required is twenty-five guineas.—T. C. C.

### How to Become a Sister-Tutor.

THE College of Nursing, 7 Henrietta Street, Cavendish Square, W. 1, awards scholarships for sister-tutors. Write to the Secretary of the College for fuller particulars. The membership fee of the College is £1 1s.—ENQUIRER.

### Private Nursing Homes in Plymouth.

APPLY to the Lady Superintendents of the two following private nursing homes: Western Counties Nurses' Co-operation, 6 Holyrood Terrace, The Hoe; The West of England Nurses' Co-operation. 4 Thorn Park Terrace, Mannamead.—FULLY-TRAINED.

### *MISCELLANEOUS.*

### The "Galvanoset" Motor Transformer.

IT is possible to obtain a Galvanoset Motor Transformer for use with your Galvanoset, with the alternating current in your district. The transformer converts the alternating current into a continuous current. The price of the transformer is £15, plus 50 per cent. increase, and can be obtained of the Medical Supply Association, 167-185 Gray's Inn Road, London, W.C.—MISS W.

### Title of Book Required.

WE think the book you refer to is "Menders of the Maimed," by Arthur Keith, which deals with the anatomical and physiological principles underlying the treatment of injuries to the muscles, nerves, bones, and joints. The publishers are Henry Frowde, Hodder and Stoughton, and the price is 16s. net. The book is copiously illustrated.—S. R. E. TEACHER.

### Automatic Time-Recorder.

AN excellent type of automatic time-recorder can be obtained from the National Time Recorder Company, Ltd., 5 Blackfriars Road, London, S.E. 1. This recorder is fitted with an eight-day pendulum clock movement and can be hung on a wall or will stand on a table. No. 1 Recorder prints the hour and exact minute, showing the distinction between A.M. and P.M. on the paper before the signature is written, which is certainly an advantage over those which print the time after the signature has been made. No. 2 also indicates automatically the day of week against each record.—Y. A. S.

### Faradisation Attachment for "Galvanoset."

IT is possible to obtain a Faradisation attachment for use with the "Galvanoset" from the Medical Supply Association, Ltd., 167-185 Gray's Inn Road, W.C. 1. The price is £1 15s., which is subject to 50 per cent. war advance.—MASSEUSE.

### Membership of the I.S.T.M.

YOU must be a holder of the Society's certificate to become a member and be nominated by members of the Society. The annual subscription is 7s. 6d. for those residing within a certain radius and 2s. 6d. for those beyond the radius. You should write to the Registrar, 157 Great Portland Street, London, W. 1, for a membership form.—W. I. P.

### Head-Mirror Lamp.

PROBABLY the best type of head-mirror lamp is an Ediswan lamp, with metal filament of helical pattern. The voltage is usually four to eight, and can be obtained either from the Ediswan Company or through most surgical-instrument makers. Messrs. Allen & Hanbury, Ltd., of Wigmore Street, Cavendish Square, W., usually keep a stock.—"PROVINCES."

